# Supplementary material for: Comorbidities, medications, depression, and physical performance measures associated with severe cognitive impairments in community-dwelling adults
Source: PLoS One. 2024 Sep 19;19(9):e0309765. doi: 10.1371/journal.pone.0309765 (PMC11412537; doi:10.1371/journal.pone.0309765)
Supplement: Attachment — Submitted filename: Response to reviewers - NA.docx [file pone.0309765.s002.docx]

Response to reviewers

Dear Editor and reviewers, thank you for your valuable feedback on our manuscript that helps us in improving the paper. We responded to each comment and provide responses with boldface across the manuscript.

**Reviewer #2:** I can see much improvement from the previous manuscript. Here are additional comments for the authors to consider:

1. **Comment:** BMI is not demographic data, but it is listed under the heading of demographics. I suggest changing the headline title to reflect the content better.

**Response: ‘**anthropometric’ has been added to the headline title and table 1

2. **Comment:** Marital status was mentioned in the method section, but it was not included in Table 1. Please include this information for completeness.

**Response: the following sentence has been added to table 1**

Marital status was dichotomized as married or divorced/widowed. We also included this in the table1.

3. **Comment:** In the introduction, the authors stated that the study aimed to examine the prevalence of severe cognitive impairment and its associated factors, later referred to as exposures. Since this is a cross-sectional study, these factors may be subject to reverse causality. It is crucial to acknowledge that the relationships identified could be due to reverse causality and should not be used definitively to determine the risk of severe cognitive impairment.

**Response: T**he following sentence has been added to the limitation of study:

The study was cross-sectional; therefore, any significant association between cognitive impairment and risk factors cannot be definitively determined from the results, rather reverse causation stands as a possibility in the observational study design.

4. **Comment:** Please clarify if there is any specific reason behind the variable choices in including the adjusted model.
5. **Response:** Thank you for your comment. The variables included in the adjusted model were selected based on their relevance in the literature and their potential association with cognitive impairment as mentioned in the introduction (line 88-99). The adjusted model for age, gender and BMI aimed to control for key factors that have been previously shown to influence cognitive outcome. To clarify this point, the second paragraph of the statistical analysis section has been amended to read as following:

Multiple binary logistic regression, odds ratio (OR), and 95% confidence interval (95% CI) were used to assess the potential associations between the risk factors and physical function measures of severe cognitive impairment. The variables included in the regression model were selected based on their relevance and potential association with cognitive impairment. The following two models were developed based on adjustments: model 1 was unadjusted, and model 2 was adjusted for age, sex, and BMI to control the key factors that have been shown to influence cognitive outcome. Previous studies employed unadjusted models [10, 41]. Missing variables were handled using case-wise deletion.

6. **Comment:** There is a missing period at line 227. Please correct this punctuation error.

**Response: Corrected**

7. **Comment:** In line 264, the steps taken to decrease measurement bias are not clearly stated. The author later describes the data validation process in lines 303 to 305, which includes data cleaning, consistency checks, sensitivity analysis, and double validation from the research assistant. In lines 363 to 365, the author discusses efforts to minimise information bias. To maintain consistency and improve the flow of the text, consider reorganising the discussion section, particularly the parts that address the limitation of measurement bias and the efforts made in the study.

**Response: T**he discussion section reorganized.

8. **Comment:** In line 267, the authors mentioned conducting sensitivity analysis. Could you specify what type of sensitivity analysis was conducted? If possible, include this information in the supplemental analysis.

**Response: Thank you for your comment. The dataset has some missing observations for some of the key predictor variables. We performed multiple imputations and compared the results.** The results for the sensitivity analysis showed similar results with the original dataset without imputation except for TUG (OR: 1.16, 95% CI [1.01, 1.34], p=0.035) that became significant predictors for severe cognitive impairment. We added the sensitivity analysis to the methods and its results to the results section for clarification. We also added to the discussion this clarification.

9. **Comment:** The authors mentioned subgroup results in the discussion section at line 269 (Severe cognitive impairment was higher in women and the 60–64-year age group.) However, the result was not confirmed in any tables or the results section. Please ensure all findings are included and clearly presented.

**Response: T**his point mentioned in the results:

**“**The prevalence of severe cognitive impairment among community-dwelling adults was 12.6% (n = 26); this was higher in women than that in men and in the 60–64-year age compared with that in the other age categories”

A clarification of the number of men/women has been added to table 1.
